# Potential applications of focused ultrasound for spinal cord diseases: a narrative review of preclinical studies

**DOI:** 10.1016/j.bas.2025.104298

**Published:** 2025-06-13

**Authors:** Ryan Nguyen, Victor Gabriel El-Hajj, Umberto Battistin, Karl J. Habashy, Victor E. Staartjes, Adrian Elmi-Terander, Jingke Zhang, Abdul Karim Ghaith

**Affiliations:** a Department of Neurological Surgery, Mayo Clinic, Rochester, MN, USA; b Department of Clinical Neuroscience, Karolinska Institutet, Stockholm, Sweden; c Department of Neurological Surgery, Northwestern University, Chicago, IL, USA; d Machine Intelligence in Clinical Neuroscience & Microsurgical Neuroanatomy (MICN) Laboratory, Department of Neurosurgery, Clinical Neuroscience Center, University Hospital Zurich, University of Zurich, Zurich, Switzerland; e Department of Radiology, Mayo Clinic, Rochester, MN, USA; f Department of Neurological Surgery, Johns Hopkins University, Baltimore, MD, USA

**Keywords:** Focused ultrasound, Blood-spinal cord barrier, Blood-brain barrier, Low-intensity focused ultrasound, High-intensity focused ultrasound

## Abstract

Focused ultrasound (FUS) technology provides unique advantages as a therapy targeting the central nervous system (CNS). We aimed to investigate and summarize the potential applications of FUS in the context of spinal cord diseases. Search strategies were created using a combination of keywords and standardized index terms. Searches were run in April 2025 in the Ovid Cochrane Central Register of Controlled Trials, EBSCO MegaFILE, Ovid Embase, Ovid Medline, Ovid PsycINFO, Scopus, and Web of Science Core Collection to retrieve all relevant studies from inception until 2024. A narrative and comprehensive summary of the current body of evidence was performed. Current preclinical studies indicate the potential use of spinal cord FUS in blood-spinal cord barrier (BSCB) disruption, neuromodulation, and inflammatory regulation following spinal cord injury. Targeted CNS drug delivery with BSCB disruption using FUS has proven promising in the context of neuro-oncology and neurotrauma. Additionally, FUS has been explored for neuromodulation in managing neuropathic pain and spasticity. FUS to the spinal cord may also provide anti-inflammatory effects and alter the local cellular response to injury. While therapeutic FUS ablation of brain structures has been extensively studied, research on similar applications within the spinal cord was less prevalent and faces multiple challenges. FUS is a highly promising technique with multiple advantages and potential applications in the treatment of spinal cord diseases. Current research efforts have shifted focus towards translational studies, while human trials are currently limited.

## Highlights

• Non-invasive focused ultrasound (FUS) for precise tissue targeting, used in neuromodulation, drug delivery, and ablation.
• FUS with microbubbles enhances drug delivery by temporarily disrupting the blood-spinal cord barrier.
• FUS modulates spinal cord function, showing promise in pain and spasticity treatments in preclinical studies.
• Difficulties in targeting through bone and safety concerns need more research for clinical use.

## 1 Introduction

Spinal pathology is a major medical and socio-economic problem that affects a significant portion of the United States population (Ryan et al., 1992). Over the last few decades, the number of patients presenting with these pathologies, including spinal cord injuries (SCI) and degenerative disk diseases, has increased across all age groups (Akter et al., 2020). Spinal imaging techniques contribute heavily to the diagnosis and management of spinal pathology by allowing characterization of the complex osseous and soft tissue components of the spine (Lee et al., 2017). The most common imaging modalities are radiography, computed tomography (CT), and magnetic resonance imaging (MRI). Despite offering two-and/or three-dimensional images of the spine, these modalities do not provide the portability and dynamic imaging ability offered by ultrasound imaging (Chin et al., 2011). These features make ultrasound particularly suitable for use in clinical scenarios such as emergency trauma assessment and image-guided non-surgical therapeutic interventions. A variety of new technologies and techniques incorporating ultrasound have been explored in the past few decades, leading to the development of therapeutic applications such as focused ultrasound (FUS).

FUS is a non-invasive therapeutic technology that uses a transducer or phased array coupled with ultrasonic energy to target deep tissue in the body without the need for incisions or radiation (Oakden et al., 2014). This allows the destruction of targeted tissue with limited damage to surrounding regions through a combination of mechanical and thermal effects (Izadifar et al., 2020). Magnetic resonance image-guided focused ultrasound (MRIgFUS) techniques further allow real-time visualization and targeting of tissue during ablation (Jolesz, 2009). In recent years, FUS has seen recognition as a clinically relevant tool with multiple purposes including drug delivery through blood-brain barrier (BBB) openings, neuromodulation in Alzheimer's and Parkinson's diseases, or thermoablation of central nervous system tumors (Lipsman et al., 2018). The technology's applications across different fields of medicine remain a major focus of current research.

The anatomic and functional complexity of the central nervous system provide a potential avenue for the precision of FUS to excel. Despite its successful clinical use in brain pathologies, FUS targeting the spinal cord remains in the preclinical stage. The majority of research performed has been in animal models. If proven safe and effective, future translational study and refinement of these techniques may reveal new paths and potential applications in human medicine (Xu et al., 2024a).

To the best of our knowledge, this is the first narrative review encompassing the present state of research into FUS therapies for spinal cord disease. We aim to briefly introduce the history and mechanisms of FUS, summarize the current preclinical literature on its use within the spinal cord, and discuss potential implementations of the technology moving forward.

## 2 Historical perspective

Lynn et al. reported the use of FUS to generate targeted thermoablation in animal tissue as early as 1942 (Lynn et al., 1942). FUS research progressed throughout the 20th century, with Fry et al. establishing its potential as a tool for therapeutic ablation of brain tissues when combined with craniotomy in 1958 (Fry, 1958; Jagannathan et al., 2009). As techniques and hardware developed further, researchers implemented FUS to treat tumors and a variety of neurological disorders including Parkinson's disease (Jagannathan et al., 2009; Meyers et al., 1959). In the current day, FUS devices have received FDA approval for the treatment of uterine fibroids, pain due to bony metastases, prostate cancer, and liver tumors. (Website) Within the brain, FUS is approved for essential tremor and symptoms of Parkinson's disease, with ongoing investigation into disruption of the blood-brain barrier, drug delivery, neuromodulation, brain tumor ablation, and other applications (Izadifar et al., 2020; Hwang et al., 2021; Darrow, 2019).

## 3 Mechanisms

The basis of ultrasound is the production of extremely high-frequency sound waves exceeding 20 kHz using a transducer placed against the body. In imaging applications, these sound waves interact with the tissues they encounter and produce reflected echoes used to construct a real-time image (Aldrich, 2007). The focusing of ultrasound beams using acoustic lenses or electronic methods allows the precise delivery of energy to generate heat and mechanical effects at a deep tissue target (Jolesz, 2009; Hwang et al., 2021). MRgFUS and other combined guidance techniques may be used to further improve user accuracy and control during the treatment. The result is a non-invasive, precise, comfortable, and relatively low-cost potential alternative to surgery or other ablative procedures (Izadifar et al., 2020).

Low-intensity (LIFU) and high-intensity (HIFU) variations of focused ultrasound have seen significant representation in neurologic research. LIFU, generally administered at less than 1 W per square centimeter, allows relatively reversible interactions with the nervous system without permanent histological changes (Darrow, 2019). This characteristic has led to significant interest in LIFU as a noninvasive approach to neuromodulation and transient disruption of the BBB or blood-spinal cord barrier (BSCB). Microbubbles (MB), miniscule gas spheres sometimes used in combination with FUS, may cavitate under targeted ultrasound stimulation to enhance the local permeability of cells to therapeutic molecules or act as direct carriers to target tissue (Sirsi and Borden, 2012; Song et al., 2018a). HIFU instead leverages intense, highly focused beams to produce localized cell death with minimal damage to the surrounding tissue. Current applications include tumor destruction and targeted ablation of other soft tissues (Izadifar et al., 2020; Jagannathan et al., 2009). While each of these technologies are heavily studied in the context of the brain, FUS to the spinal cord remains in the preclinical stage.

## 4 Methodology

An initial search was carried out using the PubMed database to identify articles related to the topic of the review. The primary search terms were “focused ultrasound” (including related terms FUS, LIFU, and HIFU), “spine”, and “spinal cord”. Preclinical studies reporting results of experiments with FUS directly targeted to the spinal cord were included. Excluded studies were those not available in English and those not available as full text publications. The identified studies were then manually searched for additional relevant citations.

**Fig. 1:**
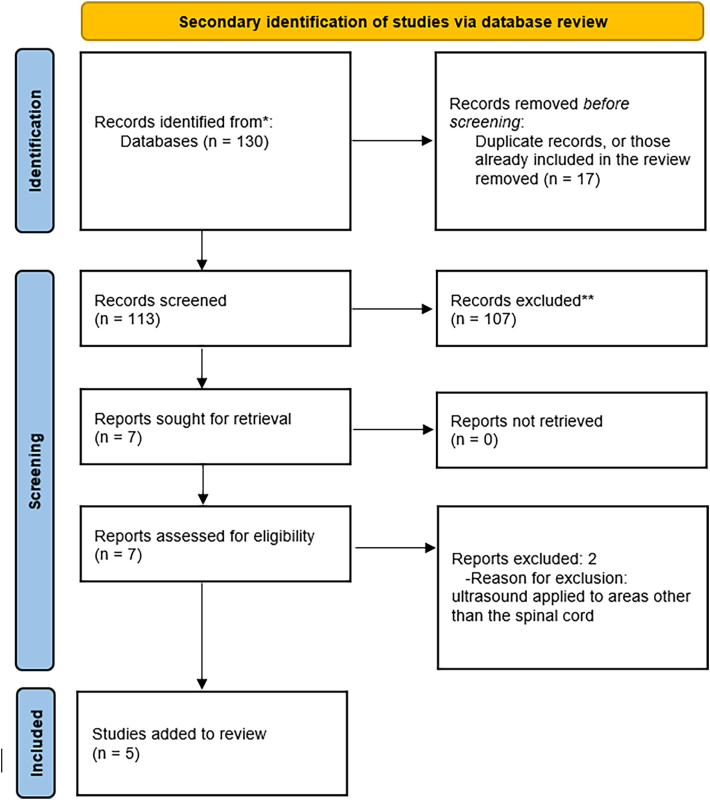
PRISMA flowchart.

A supplementary structured search of the PubMed database using the Boolean operator string (“focused ultrasound" [Title/Abstract] OR “LIFU" [Title/Abstract] OR “HIFU" [Title/Abstract]) AND (“spinal" [Title/Abstract] OR “cord" [Title/Abstract] OR “spine" [Title/Abstract]) was performed to identify further studies which had not been identified by the primary search. The article screening approach applied to this component of the search is documented in Fig. 1.

### 4.1 Blood-spinal cord barrier opening and treatment delivery

The BBB and BSCB represent key obstacles in drug delivery to the central nervous system (CNS). A useful application of FUS is the transient, selective disruption of these barriers to facilitate molecule delivery, particularly when combined with MB techniques (Bhimreddy et al., 2023; Fletcher et al., 2020a). In the current preclinical literature, this has been leveraged to study potential therapies for spinal cord disease (Table 1).

**Table 1:** Pre-clinical focused ultrasound blood−spinal cord barrier opening studies.

Study	Year	Experimental Subject	N	Target	MB Dose	Laminectomy	Frequency [MHz]	Total time [s]	Peak Pressure [MPa]	Post-FUS spinal cord damage
Shimamura et al.	2005	Rat (260–300 g)	40	T9-T10	25 μl Optison	Yes	1	5 s	0.05–0.12	No
Takahashi et al.	2007	Mice (7–8 weeks)	60	Thoracic	<10 μl Optison	No	0.95	60 s	0.2	No
Wachsmuth et al.	2009	Rat (300–500 g)	31	Cervical spine	0.043 μl/kg Definity	No	1.08	300 s	0.28–0.55	No
Ando et al.	2011	Rat (180–270 g)	40	T10	–	Yes	Photomechanical	–	45/5	No
			20						135/3	Yes
Ando et al.	2012	Rat (180–270 g)	40	T10	–	Yes	Photomechanical	–	50/8	No
Oakden et al.	2014	Rat (250–400 g)	19	Cervical spine	0.02–0.04 ml/kg Definity	no	1.114	300 s	0.72–0.88	Yes
Oakden et al.	2015	Rat (250–400 g)	16	Cervical spine	0.02–0.04 ml/kg Definity	no	1.114	300 s	0.88	Yes
Weber-Adrian et al.	2015	Rat (∼300 g)	12	Cervical spine	0.02 ml/kg Definity	No	1.114	300 s	0.4	Yes
Song et al.	2017	Rat (180–200 g)	158	T10	50 μg, nanobubble	Yes	1?	300 s/day for 3 days	0.27	Yes
Payne et al.	2017	Rat (200–235 g)	14	Cervical + Thoracic	0.02–0.06 ml/kg Optison	No	0.94	180 s	1.0–2.1	Yes
Song et al.	2018	Rat (220–250 g)	92	T10	50 μg, nanobubble	Yes	1?	300 s/12h for 3 days	0.35	No
O'Reilly et al.	2018	Rat (∼200 g)	18	Tumour regions in cord	0.02 ml/kg Definity	No	0.552	120 s	0.22–0.43	No
Montero et al.	2019	Rabbit (3.0–4.3 kg)	15	T4, T7, T11, L3	0.2 ml/kg SonoVue	Yes	1.1	150 s	0.3–0.8	Variable
Fletcher et al.	2019	Rat (173–314 g)	7	Thoracic	0.02 ml/kg Definity	No	0.514	120 s	0.46–0.69	Yes
			7							
Shah et al.	2020	Mouse (∼22 g)	51	Low thorax - Upper lumbar	0.02–0.04 mL/kg Definity	No	1.68	120 s	0.62–0.64	No
Fletcher et al.	2020	Pigs (32–40 kg)	4	–	0.005–0.01 mL/kg Definity	No	0.486	120 s	1.0–4.0	Yes
			6					300 s	1.0–2.1	
Smith et al.	2021	Rat (220–280 g)	13	T9 and T12	0.02 mL/kg Definity	No	0.58	120 s	0.27–0.45	No
Cross et al.	2021	Rat (200–300 g)	24	T8 - T10	0.2 mL/kg Optison	Yes	0.94	180 s	1.0–2.1	–
Fletcher et al.	2021	Rat (199–573 g)	4	Low thoracic	0.02 mL/kg Definity	No	0.514	120 s	0.3–0.49	Yes
			4					300 s	0.42	
			4					300 s	0.42	
			4					120 s	0.42	
			4					120 s	0.42	
Bhimreddy et al.	2023	Rat (250g avg)	5	Thoracic	0.2 mL Lumason (Bracco)	Yes	0.25	300 s	0.47	No

Our review included 20 preclinical studies on the use of FUS for blood-spinal cord barrier opening (BSCBo). A notable trend is the presence of spinal cord bleeding, particularly in older studies from the mid-twentieth century (Bhimreddy et al., 2023; Fletcher et al., 2020a; Cross et al., 2021; Fletcher et al., 2020b; Xu and O'Reilly, 2018; Song et al., 2018b; Song et al., 2017; Payne et al., 2017; Fletcher et al., 2021; Shimamura et al., 2005; Takahashi et al., 2007; Ando et al., 2012; Ando et al., 2011; Oakden et al., 2015; Weber-Adrian et al., 2015; Song et al., 2023). The first study of BSCBo was published in 2005. A key finding from these studies is that the minimum and mean pressure damage thresholds do not accurately predict vascular or spinal cord damage. This is primarily due to the use of pre-formed MB, which can produce mechanical effects at lower pressures. The studies found that peak negative pressure, MB dose, and gas volume were significantly associated with damage thresholds. Porcine models presented additional challenges, requiring higher pressures, and exhibiting variable BSCBo efficacy depending on spinal level. However, they were preferred in cardiovascular research for their physiological similarity to humans. Histological examinations frequently revealed varying degrees of tissue damage. Despite multiple studies showing damage, only one reported ultrasound-induced paralysis, which could be linked to cold MB. Several MB types were used due to their distinct contrast properties. The ultrasound frequencies and peak pressures varied significantly between studies, reflecting diverse research objectives and applications.

SCI may cause significant burdens to patients, and current treatment options offer limited hope of functional recovery. The growing field of gene therapy offers a broad array of possible targets, but an ideal vector for SCI therapy has not been identified. A 2017 study by Song et al. combined ultrasound and poly (lactic-co-glycolic acid) (PLGA) nanobubbles (NB) to deliver nerve growth factor (NGF) to the site of injury in 108 rats with induced thoracic SCI (Song et al., 2017). NGF is a well-characterized neurotrophic factor thought to play neuroprotective and possibly regenerative roles in the damaged spinal cord (Zhang et al., 2014). The rats were randomly split into four groups: 1) an control group receiving only a normal saline injection to the tail vein; 2) a group receiving an NGF plasmid + NB solution injection to the tail vein; 3) a group receiving an NGF plasmid solution injection followed by US; 4) a group receiving an NGF plasmid + NB solution injection followed by US. Injections occurred every 12 h for 3 days, with US for 5 min. The group receiving NGF, NB, and US displayed successful transfection of the NGF gene with significantly greater neuronal survival, decreased apoptosis, and improved Basso–Beattie–Bresnahan (BBB) locomotor scores at up to 28-day follow-up, representing the prospect of short-term functional recovery compared to the other groups. Song et al. acknowledged methodological limitations including the short period of follow-up and inability to administer ultrasound to the spinal cord without exposure through laminectomy.

A second study by Song et al. elaborated upon the US/MB gene delivery technique by incorporating LIFU and cationic nanobubbles (CNBs) (Song et al., 2018b). The positive charge possessed by CNBs reduce the repulsion between traditionally neutral or negatively charged MB and nucleic acids, improving DNA binding and efficiency of gene transfer (Sun et al., 2013). Plasmids promoting expression of brain-derived neurotrophic factor (BDNF), a potent neurotrophic factor that promotes neuronal growth, differentiation, survival, and synaptogenesis, were loaded into the CNBs. Antibodies against microtubule-associated protein 2 (MAP-2), a neuronal molecular marker, were conjugated to improve the specificity of targeting. The results indicated a significant increase of BDNF expression in treated rats. Rats transfected with this method displayed decreased histological injury, limited loss of neurons, and improved BBB locomotor scores upon extended observation through one month. These findings provide further preliminary support for the potential of FUS/MB in SCI therapy.

**Fig. 2:**
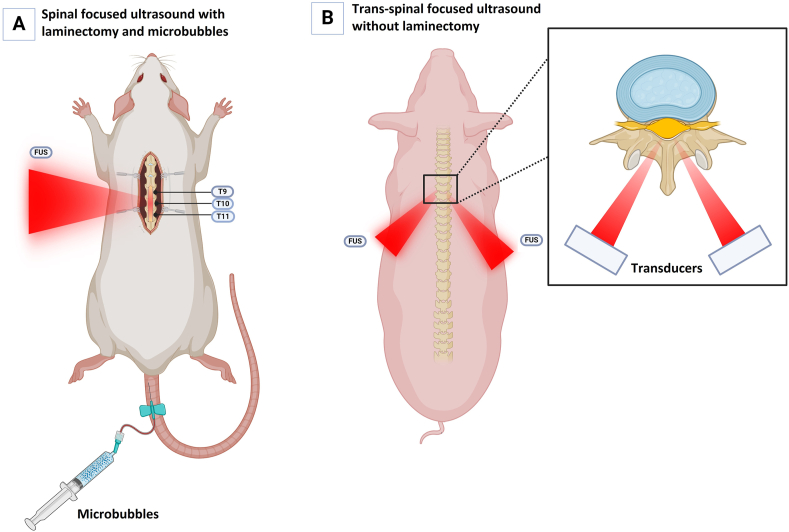
Focused ultrasound (FUS) application in preclinical models: Surgical laminectomy in rats (A) and noninvasive dual-aperture transvertebral FUS in Pigs (B).

Due to the difficulty of ultrasound delivery through bone, laminectomy is a mainstay of FUS approaches to the spinal cord. While this may be practical in treatment of localized disease, non-invasive options to bypass the spine are required to apply FUS in disseminated spinal conditions and represent a major challenge in clinical translation (Fletcher et al., 2020b). Furthermore, existing trans-spinal focused ultrasound (tsFUS) techniques, which pass ultrasound directly through bony elements of the spine and have seen testing in rats, may not be suitable in the larger, denser human spine (Fletcher et al., 2020b). Fletcher et al. developed a dual-aperture approach employing phase key shifting and short burst exposure alongside MBs to address this issue (Fletcher and O'Reilly, 2018). This method employs two separate transducers to produce a region of focus within the spinal canal. Initial testing with ex vivo thoracic human vertebrae were successful in producing a controlled focus without the generation of potentially hazardous standing waves (Fletcher and O'Reilly, 2018). In a porcine model, BSCBo was assessed through intravenous injection of Evans blue dye with 1.5 h of time to circulate (Fletcher et al., 2020b). Successful dye extravasation was observed in 16 of 24 treatment locations throughout the lower thoracic and upper lumbar spine. Notably, using sinusoidal pulse exposure led to consistent surface hemorrhage to the cord alongside extensive damage on histology. When instead employing short burst exposure with phase key shifting, 3/24 cases displayed macroscopic damage to the spinal cord and 4/24 displayed very minor damage on histology, particularly to the central gray matter. Ultimately, the noninvasive short burst exposure and phase key shifting approach was successfully used to open the BSCB through the intact porcine spine. This finding has major implications for the future of spinal cord FUS to the spinal cord and presents a set of principles that may decrease the need for invasive approaches. Fig. 2A illustrates an example of FUS following surgical laminectomy in a rat model. It depicts the use of FUS on vertebral levels T9, T10, and T11 following surgical exposure and closure of the spinal cord. A syringe attached to the rat's tail vein suggests the administration of MB before the sonication. Fig. 2B shows a noninvasive dual aperture transvertebral FUS technique in a pig model. The image depicts two ultrasound transducers focused through the vertebrae on a specific area of the spinal cord without any laminectomy. The inset shows a cross-sectional view of how the FUS beams converge on the spinal cord.

Metastasis to the leptomeninges (LM) is a rare but dangerous complication arising from a variety of cancers. Within the brain and spine, LM is often diagnosed late into development. As a result, management is generally centered around palliative radiotherapy (O'Reilly et al., 2018; Nguyen et al., 2023). A continual challenge in their treatment is the penetration of the BBB or BSCB to reach LM with systemic therapy. O'Reilly et al. hypothesized FUS + MB could facilitate delivery of trastuzumab to the spinal cord and improve outcomes in rats with induced LM (O'Reilly et al., 2018). A total of 18 athymic rats were implanted with HER2-expressing breast cancer cells in the thoracic subarachnoid space and split into three groups: 1) no treatment; 2) intravenous trastuzumab; 3) intravenous trastuzumab with FUS and MB. Treatment was administered weekly for three weeks total. The FUS + MB + trastuzumab group displayed suppressed tumor growth and reduced tumor burden on MRI with no associated improvement to overall survival. This small feasibility trial indicates FUS with MB may be a future target in the field of spinal cord tumor therapy.

Smith et al. published a follow-up study in 2021 with the intention of clarifying the delivery patterns and distribution of trastuzumab using FUS + MB in both healthy and LM rats (Smith et al., 2021). Quantification of trastuzumab was achieved through human IgG enzyme-linked immunosorbent assay (ELISA) and immunohistochemical analysis. In healthy rats 2 h after treatment, the authors observed localized trastuzumab levels at 12 ± 5 times the level in control tissue. This level returned to baseline at the 24-h assessment. In 3 LM rats receiving the treatment, trastuzumab was quantified at equal or greater levels in tumor samples compared to control tissue. These preclinical findings suggest FUS may function to temporarily increase BSCB permeability, facilitating drug delivery and providing a similar effect to that seen in the brain.

**Fig. 3:**
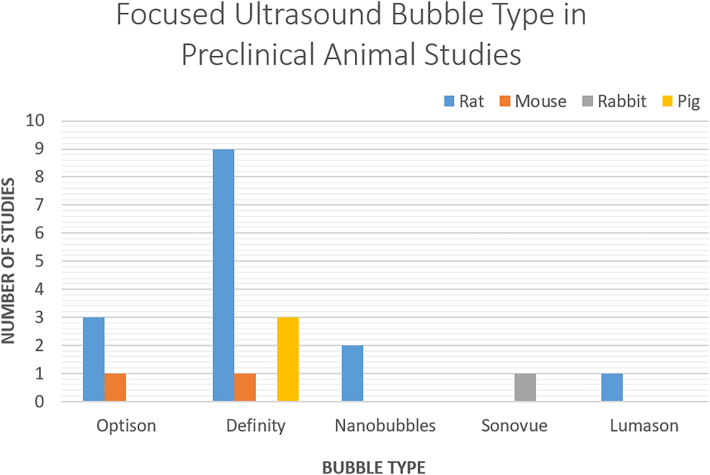
Usage of different types of bubbles in preclinical studies across various animal models.

Fig. 3 is a bar graph that depicts the use of various types of MB in preclinical FUS studies. It reveals a significant reliance on Definity microbubbles, particularly in rat studies. NB are also used extensively, particularly in rat models, most likely due to their improved precisely target deep tissues. In contrast, Optison and Lumason are less commonly used, indicating a narrower application range or possibly more specific research objectives. The limited use of SonoVue is noticeable, with only a few studies using this bubble type.

### 4.2 Neuromodulation

Our review included ten preclinical studies on FUS neuromodulation in the spinal cord (Table 2). (Song et al., 2023; Takagi et al., 1960; Tsehay et al., 2023; Kim et al., 2022; Liao et al., 2021a, 2021b, 2022; Ballantine et al., 1960) The first FUS neuromodulation studies were conducted in 1960, and demonstrated reversible effects on spinal cord reflexes in cats and toads (Takagi et al., 1960; Ballantine et al., 1960). Key findings show that FUS can enhance and suppress spinal cord reflexes and function, with effects influenced by amplitude, pressure, and treatment duration. A variability in neuromodulatory responses was noted, which was influenced by differences in the targeted spinal regions and ultrasound parameters used in various studies. Notably, chronic FUS applications in rodent models improved spasticity and nerve function after several weeks of treatment. Tsehay et al. and Song et al. further demonstrated that FUS can modulate motor-evoked and H-reflex potentials in rodents (Tsehay et al., 2023).

**Table 2:** Pre-clinical focused ultrasound spinal cord neuromodulation studies.

Study	Year	Experimental Subject	N	Target	Laminectomy	Freq. [MHz]	Total Time [s]	Peak Pressure [MPa]	Post-FUS spinal cord damage	Neuromodulatory Effects
Takagi et al.	1960	Toad	1	–	–	1	1 s	0.3–1.4	NA	Spontaneous discharges, eventual inhibition
Ballantine,et al.	1960	Cat	11	L7/S1	Yes	2.7	51–555 s	3.2	No	Reversible enhancement and depression (separately) of spinal cord reflex
			5				3 s	3.2	No	Reversible enhancement of spinal cord reflex
			1				4 s	11	Yes	Reversible enhancement of spinal cord reflex
			9				≤3 s	6.9	No	Reversible enhancement of spinal cord reflex
			14				≥5 s	6.9	Yes	Reversible enhancement of spinal cord reflex
Liao et al.	2021	Rat (220–300 g)	24	L4/L5	No	4	20 s	0.5–1.5	No	Soleus recruitment proportional to pressure
			24				20 s	1.5–3.0	Yes	Recruitment still increased with damaging pressures
Liao et al.	2021	Rat (220–300 g)	40	L4/L5	No	4	1200 s/day, 4 weeks	0.65	No	**Improvement in a peripheral nerve injury model**
Liao et al.	2022	Rat (220–300 g)	30	T8-T10	Yes	4	1200 s/day, 4 weeks	0.65	No	**Improvement of spasticity in an SCI model**
Wang et al.	2022	Rat (200–300 g)	18	T9	Yes	1	1200 s/day, 4 weeks	0.68	No	**Improvement of spasticity in an SCI model**
Kim et al.	2022	Mouse (∼20 g)	50	T12	No	3	200 s	0.8–2.2	No	Depressive effect (increasing with amplitude) at one location, enhancing effect (at high amplitude) at second location
			50				200 s	1.4–2.2	No	No ascending effect at either location
Hong et al.	2022	Rat (240 g±40 g)	29	T10	Yes	1	300 s	0.65	Yes	**Improvement in BBB score relative to sham treatment**
			25				300 s	0.22	Yes	**No difference in recovery relative to sham treatment**
Tsehay et al.	2023	Rat (250–300 g)	10	T11/T12	Yes	0.5	300–600 s	0.042	No	Sonications generated transient suppression of MEP signals
Song et al.	2023	Rat (250–450 g)	35	T9, T13, L5, S2	No	1.1	60 s	0.84–1.72	No	Suppression of H-reflex amplitude, decreased latency
			35				60 s	1.48	No	Slight enhancement of H-reflex amplitude, decreased latency

Between 2021 and 2022, Liao et al. reported a trio of studies evaluating the use of LIFU as a neuromodulatory tool in the murine spinal cord (Smith et al., 2021; Colloca et al., 2017). These findings represent some of the first modern steps towards therapeutic FUS neuromodulation in the spinal cord. The first study investigated the use of LIFU to treat neuropathic pain in rats with induced peripheral nerve injury (PNI) impacting the L4-L5 distribution (Liao et al., 2021a). Chronic neuropathic pain may be debilitating for patients and is difficult to effectively treat. Current pharmacological options are limited in efficacy and often associated with burdensome adverse effects or the risk of addiction (Colloca et al., 2017). The effect of LIFU in the study was measured using the 50 % paw withdrawal threshold (PWT_50_) to model allodynia. In addition, the authors quantified the spinal cord expression of multiple markers believed to participate in neuropathic pain development following PNI. PWT_50_ decreased initially after PNI induction in both the treatment and control groups but increased significantly following 4 weeks of LIFU. Potassium chloride cotransporter 2 (KCC_2_), a marker downregulated in the development of NP, displayed a similar downregulation after the injury followed by increased expression after LIFU stimulation. These findings show the ability of LIFU to alleviate neuropathic pain in rats with PNI, possibly through upregulation of KCC_2_ in spinal neurons.

The second study by Liao et al. aimed to quantify the relationship between LIFU intensity and resulting stimulation or damage to spinal cord neural circuits (Liao et al., 2021b). Varying intensities of LIFU were applied to the L4-L5 segment and electromyography was used to record activation of the soleus muscle. Next, a battery of tests including electromyography, enzyme-linked immunosorbent assays, the BBB locomotor scale, and immunohistochemical staining were performed to assess the safety of the applied frequencies. LIFU intensity greater than 1.0 MPa (MPa) induced significant activation of the soleus. Stimulation with an intensity of 3.0 MPa produced significant spinal cord damage and decreased somatosensory evoked potential amplitude. A threshold of <1.5 MPa neurostimulation was suggested, as this intensity was able to induce muscle recruitment without measurable LIFU-associated damage.

In addition to its stimulatory effects, FUS has the potential to inhibit CNS activity. A third study published by Liao et al., in 2022 assessed the impact of LIFU on spasticity in rats with induced SCI (Liao et al., 2022). When compared to a sham control group and FUS^−^ group, rats receiving 4 weeks of FUS stimulation displayed significantly reduced spasticity behavior and significantly increased expression of spinal cord KCC_2_. Wang et al. reported similar results in their 2023 article, observing increased spasticity at 2 weeks post-SCI followed by significant improvement in rats treated with LIFU (Wang et al., 2023). Notably, SCI without LIFU was associated with significantly increased growth associated protein 43 (Gap43) expression compared to the sham group, with a corresponding decrease in expression observed with LIFU treatment. Gap43 may play an important role in the post-SCI microenvironment and could play an important regulatory role in SCI-related spasticity. A study by Kim et al. evaluated the effect of tsFUS on mice with Harmaline-induced essential tremor. Statistically significant tremor suppression was observed after 13 s of stimulation over T12, lasting up to 23 s once the tsFUS ended. tsFUS to the L3 region instead amplified muscle signals. No histological damage was observed, with pressures up to 2.2 MPa applied (Kim et al., 2022). Song et al. measured the modulatory effects of 1 min of tsFUS on rat spinal reflexes (Song et al., 2023). Reversible suppression of the sciatic nerve Hoffman reflex was observed and noted to be acoustic pressure-, pulse-repetition frequency-, and spinal level-dependent. The acoustic pressure of 1.48 MPa used in the study did not cause any measurable tissue damage. These studies offer a baseline for future research into FUS-centric management of spasticity and motor disorders. While the significant potential of FUS neuromodulation is clear, additional studies must further optimize the safety, stimulation parameters, and targeting of FUS in animal models before translation into clinical study is considered.

### 4.3 Anti-inflammatory effects

The puzzle of managing SCI includes multiple time-sensitive components. The primary mechanical insult is typically followed by a secondary wave of local inflammatory activity which worsens the injury and promotes BSCB disruption (Freyermuth-Trujillo et al., 2022). Immune cells are continuously recruited to the area leading to regional remodeling and deposition of scar tissue. Components of this toxic inflammatory response are believed to persist for months to the patient's lifetime.

Hong et al. developed a set of US stimulation protocols to assess the impact of pulsed LIFU on the inflammatory response following induced spinal contusion in a rat model (Hong et al., 2022). Following SCI, 105 rats received T10 dorsal laminectomy and were assigned to sham or US pulses at 1 MHz and rates of either 5 % (SCIU5, hypothesized to have an inhibitory effect on neuronal activity) or 40 % (SCIU40, hypothesized to result in greater neuronal stimulation or excitation) duty cycles with the same overall acoustic intensity of 0.8 W/cm^2^. These treatments were administered for 5 min per day on days 2, 3, and 4 following the injury. BBB score and inflammatory markers were then compared between the groups during the acute (<1 week) and chronic (1–8 weeks) phases of spinal cord inflammation.

When compared to the sham group, rats treated with the SCIU5 protocol had significantly greater BBB scores at 5 days (6.8 ± 2.5 vs. 4.3 ± 1.5, p = 0.007) and 7 days (13.3 ± 2.5 vs. 9.9 ± 2.6, p = 0.0002). The effect of SCIU5 treatment on the rats’ BBB scores normalized to the level of the sham group over the full 8-week period of the study, but measures of hindlimb motor function were slightly improved. A similar effect was not seen in the SCIU40 group, which overall displayed a comparable rate of motor recovery to the sham group. Furthermore, the SCIU5 group had decreased inflammatory marker activity, including lower levels of local TNF-α, ED-1, and iNOS representing macrophage recruitment and activation. On histological analysis, SCIU5 treatment increased local tissue density and reduced the presence of βIII-tubulin + FJC + DAPI triple-positive cells, indicating lower rates of neuronal cell degeneration. The spinal cord lesion cavity area and quantified myelination were similar between the SCIU5 and sham groups. These preliminary findings support the potential of low-duty-cycle LIFU as a future anti-inflammatory intervention in both acute and chronic SCI. Further experimentation with variable US parameters could provide more insight regarding the dynamic relationship between US and SCI inflammation and guide researchers toward more nuanced therapeutic protocols.

Neuropathic pain following SCI or peripheral nerve injury is thought to be partially mediated by chronic neuronal inflammation. A recent study by Song et al. applied LIFU neuromodulation techniques to rats with induced chronic constriction injury with the goal of comparing intraspinal microglial response and mechanical sensitivity as measured by the von Frey Threshold (vFT) (Song et al., 2025). 1.1 MHz pulses with a 40 % duty cycle were applied to a lateral aspect of the spinal cord at the L5 vertebra for 3 min daily on days 4, 5, and 6 following ipsilateral induced sciatic nerve constriction injury. Interestingly, the FUS-treated regions of spinal cord reduced microglial quantity and activation compared to untreated regions. FUS-treated animals displayed decreased mechanical sensitivity compared to the sham group across the full 23-day observation period.

The reported anti-inflammatory effects in murine neurological injury models are interesting but remain limited relative to the likely chronic nature of SCI-associated inflammation and scarring. The effect of FUS on other components of the complex spinal immune response remains unclear. Future research to determine whether FUS can safely and effectively influence the SCI microenvironment over longer treatment periods should be performed.

### 4.4 Focal ablation

FUS ablation of brain structures has been studied extensively. The ability of ultrasound to alter and destroy CNS tissue was first reported in the 1900s (Fry, 1958; Jagannathan et al., 2009; Ballantine et al., 1956). While this application continues to receive significant focus, little information is currently available regarding therapeutic FUS ablation of the spinal cord. Studies of FUS targeting adjacent structures such as the intervertebral discs, facet joints, and nearby sensory nerves have shown promising preliminary results (Qiao et al., 2019; Tiegs-Heiden et al., 2023; Kaye et al., 2016). However, tumors within or prohibitively near the spinal cord are frequently excluded from such studies due to the risk of thermal nervous damage (particularly in the absence of insulation for nervous tissue), and preclinical research regarding ablative spinal cord FUS approaches is limited (Xu et al., 2024a; Bing et al., 2018; Tsoumakidou et al., 2016; Chan et al., 2017; Huisman et al., 2014). A 2024 safety review by Xu et al. reports on numerous historical studies performed in animal models between 1950 and 1999 to understand the context of direct, irreversible spinal cord FUS exposure (Xu et al., 2024a). Xu et al. were able to characterize the estimated relationship between ultrasound exposure and spinal cord damage, providing useful thresholds to guide safe practices in future preclinical studies. Despite this significant advancement and concurrent improvements in FUS technology, the expansion of ablative FUS to the spinal cord represents a challenging domain to traverse with little study in recent decades. Additional high-quality preclinical research is required to better understand the technology's potential.

### 4.5 Current challenges and future directions

As the body of preclinical literature surrounding spinal cord FUS continues to develop, several important challenges have arisen. Notably, penetration of the beam through the complex bony vertebral structures is difficult. While many current studies achieve ultrasound focus to the spinal cord using laminectomy, this removes the benefits of FUS as a noninvasive therapy and decreases its clinical applicability. Future improvements to noninvasive transvertebral ultrasound could be central to addressing this issue (Fletcher et al., 2020b; Fletcher and O'Reilly, 2018; Xu and O'Reilly, 2022; Martin et al., 2024).

The safety of therapeutic spinal cord FUS also remains unclear. Ultrasound produces a variety of biophysical effects when applied to tissue, not all of which are fully understood. Despite the many potential benefits of FUS to treat neurological conditions, existing applications are not without risk (Pérez-Neri et al., 2022). Recent studies have aimed to address safety concerns including spinal heating and other barriers to therapeutic use of FUS (Xu et al., 2024b; Olinger et al., 2023). The body of dedicated spinal FUS safety research is still growing and should continue to be addressed through additional preclinical study.

FUS in the spinal cord is a growing field with significant potential. Future research should continue to explore therapeutic possibilities and the mechanism of action in different pathologies while including longer follow-up periods to better understand the long-term safety profile. Technological development and optimization are also required to facilitate the translation of these findings into clinical studies given the practical and biological differences between animal and human use.

## 5 Conclusion

FUS is a highly promising technique with multiple advantages and potential applications in the treatment of spinal cord diseases. This review of the literature discusses current preclinical research into possible anti-inflammatory, neuromodulatory, and BSCB-disruptive FUS technologies. These effects, while intriguing, are not fully characterized. Furthermore, the optimal protocols and long-term safety of FUS have not been established. Current research efforts have shifted focus towards translational studies, while human trials remain limited.

## Ethical approval

All ethical guidelines were adhered to during conception of this work. As this work was a systematic review of the literature, IRB approval was not sought.

## Funding

This research received no funding, and none of the researchers received any compensation relevant to this work. Dr. Staartjes is supported by the Gottfried und Julia Bangerter-Rhyner Stiftung, unrelated to this work.

## Declaration of interests

None of the authors declare any conflict of interest.
